# Hospital Meetings, &c.

**Published:** 1899-07-01

**Authors:** 


### HOSPITAL MEETINGS, &C.

### THE ROYAL LONDON OPHTHALMIC HOSPITAL.

Opening of New Buildings.

On Tuesday afternoon their Royal Highnesses the Duke and Duchess of York, who were attended by Sir Charles Cust and Lady Katherine Coke, opened the new buildings of the Royal London Ophthalmic Hospital in City Road. The Royal visitors arrived about half-past three, and were received by Sir John Lubbock, M.P. (president of the hospital), and Mr. H. P. Sturgis (chairman of the Committee of Management). Mr. Keith D. Young and Mr. Henry Hall (the architects) were presented, and handed to the Duke a handsome engraved and embossed silver gilt key, with which the entrance door was duly opened. Before inspecting the building the members of the Committee of Management, Miss Ada Robinson, the matron, and Mr. R. J. Bland (secretary) were presented. Very effective floral decorations were arranged both in the corridors and in the waiting hall of the out-patient department, where the ceremony of the opening took place, and on arriving at the platform the Duchess received a beautiful bouquet of roses from little Miss Tweedy, daughter of Mr. J. Tweedy, F.R.C.S., one of the surgeons of the hospital. Amongst the guests on the platform were: The Right Hon. Sir John Lubbock, Bart. (president), and Lady Lubbock, the Right Hon. the Lord Mayor (vice-president *ex-officio*) and the Lady Mayoress, Mr. H. P. Sturgis (chairman) and Mrs. Sturgis, the Right Rev. the Bishop of Islington and Mrs. Turner, the Rev. Prebendary Whittington (chaplain), Mr. J. Lea Smith (trustee), Mr. Keith D. Young and Mr. Henry Hall (architects). Other visitors were: Mr. Alderman and Sheriff Alliston, Lieut.-Colonel and Sheriff Clifford Probyn and Mrs. Clifford Probyn, the Right Hon. Sir Bernhard Samuelson, Bart., and Lady Samuelson, Sir John Whittaker Ellis, Bart., and Lady Whittaker Ellis, Sir George Faudel-Phillips, Bart., and Lady Faudel-Phillips, Sir Thomas J. Lipton, Sir Squire Bancroft and Lady Bancroft, and the Rev. Dr. Hermann Adler and Mrs. Adler.

Proceedings were opened by the Bishop of Islington, and welcoming speeches followed from Mr. Sturgis and Sir John Lubbock, the former explaining the financial position of the hospital and the need of funds to carry on its increased work.

In reply, the Duke of York said: Sir John Lubbock, Mr. Sturgis, ladies, and gentlemen,—We are very grateful to Sir John Lubbock for the kind words he has used with regard to our coming here to-day, and we have been very much interested in what we have heard from Mr. Sturgis. I thank you all in the Duchess's name as well as in my own for the very kind reception you have given us. It is especially a pleasure to the Duchess and myself to come here to-day, as my father laid the foundation-stone of this new building in 1897, and we are thus completing the work, so to speak, which he inaugurated. As Mr. Sturgis told us just now, the number of patients increased so enormously that the old building was found quite inadequate to the needs, and the committee were compelled to seek a larger site for their new institution. If I may be allowed to do so, I should like to congratulate the architects, Messrs. Young and Hall, on the excellent results of their labours, and I also wish to congratulate the committee, the medical staff, and the governing body of the new hospital, which has been designed and equipped according to the most modern requirements. The cost of the maintenance of this new building, which covers three-quarters of an acre, will be, I fear, very heavy, but I am sure the committee deserve the generous support of the charitable public to enable them to continue the useful work which has been so ably carried out in the hospital for nearly a century. I can only say that I trust the public will come forward and prevent the institution getting into debt. I may also say that the present attendance at the hospital is about 400 daily. The operation of removing cataract from the eyes of young children and saving them from becoming blind was originated at the Moorfields Hospital. I have now much pleasure in declaring this new building to be opened, and the Duchess joins with me in wishing the Royal London Ophthalmic Hospital continued prosperity in its new building, and a long career in its great and important work.

The new hospital, which contains 140 beds, is built on an irregular-shaped piece of land, some 35,000 ft. in area, with frontages to City Road, Cayton Street, and Peerless Street. There are extensive out-patient departments, admirably planned and arranged for the control of large numbers of patients, on the ground floor, and above the plan consists of three distinct blocks. The ground floor of the City Road block is devoted to administrative offices, and the first floor to accommodation for the staff and the matron. On the second floor are an operation theatre, with anæsthetic and recovery rooms, &c., two small wards, sisters' rooms, and nurses' sitting-room; above, again, come kitchens and offices, nurses' dining-room, and servants' quarters; the nurses' bedrooms being provided on the corresponding floor of the intermediate block, and special wards for sick nurses on a fourth floor, entirely isolated. The wards are large and wide, with teak floors and Teale grates; corridors, &c., are warmed by hot water radiators; electric light has been laid on throughout the building, and generally the most approved devices of modern hospital architecture have been carried out under the direction of Messrs. Keith Young and Hall, the architects. The furnishing and fitting has been done by Messrs. Hampton, of Pall Mall.

The old buildings at Moorfields have sold for £78,000, the new hospital costing about £80,000. Increased funds will now be urgently needed to maintain this great institution in the fullest efficiency, and the committee appeal for £50,000 to secure the ground rent during the term of the lease—99 years.

### THE LONDON HOMŒOPATHIC HOSPITAL.—JUBILEE CELEBRATION.

A festival dinner was held at the Hotel Cecil on Wednesday, June 21st, to celebrate the fiftieth year of the London Homœopathic Hospital. The Earl Cawdor (treasurer and vice-president) was to have presided, but was prevented by ill-health from fulfilling his engagement. His lordship's place was taken by Mr. J. P. Stilwell, the chairman of the Board of Management.

In giving "The Queen and the Royal Family," the Chairman mentioned that in 1897 the hospital received a grant of £245 from the Prince of Wales's Fund, and in 1898 £200, for which assistance they were very grateful. Gratitude, however, was a sense of favours to come, and they looked forward to a further grant this year. The representatives of the Fund who visited the hospital were not only satisfied with all they saw, but were very much pleased and went away with the very best feelings towards the institution. (Applause.)

The toast having been duly honoured,

The Chairman gave "The London Homœopathic Hospital: Its Founders and First Friends." He first expressed regret at the absence, through indisposition, of the Earl Cawdor, who, he said, had been a worthy supporter of the hospital for many years. With his letter of apology his lordship sent a cheque for £100, expressing the hope that they might raise sufficient that night to place the finances of the hospital on a thoroughly satisfactory footing. Sir Henry Tyler also wrote regretting he could not be present. Turning more particularly to the toast, which he described as the toast of the evening, the Chairman (who was very indistinctly heard where the representatives of the Press were placed) was understood to say that among the earliest benefactors were Dr. Quin. Lord Ebury was chairman in 1854-88, and president in 1888. Nathaniel Barton was their treasurer from the foundation of the hospital in 1849 till 1868. Major William Vaughan Morgan, a subscriber from the date of removal to Ormond Street, did more for the consolidation, development, and financial progress of the hospital than any supporter of that period. In further remarks the Chairman referred to the interest taken in the hospital by the late Duchess of Teck, and concluded by appealing to those present for liberal gifts.

The toast was accorded an enthusiastic reception, and the Chairman then added some observations with reference to the institution. He did not want to trouble the company with statistics, but he thought they would be interested to learn that, whereas in its first year 156 in-patients were treated at the hospital, 1,111 were treated in the forty-ninth year; and whereas the out-patients treated in 1850 numbered 1,546, 18,551 were treated in 1898. He could add nothing to those figures to show how the work of the hospital was appreciated. As to the call they were making that night, and the cause of it: having built their new hospital they were doubling the number of beds. They had a very expensive hospital, which had been paid for, thanks to the generosity of their supporters. Considering that the annual income had not increased in the same ratio as their work had increased, they had been obliged to ask their friends that evening for £15,000. The question was whether they should continue the present efficiency of the hospital, or whether they should "draw in their horns" and do less. He hoped they would be supported, and that the good work being carried on would be continued to the full. (Applause.) It was the object of the board of management, wherever possible, to save expense. In such a large hospital, however, they could not materially draw in their expenditure without diminishing efficiency. (Applause.)

Mr. Sydney Gedge, M.P., in an able speech, sparkling with humour, submitted "The Board of Management and the Medical Staff." On the previous day he had had the great pleasure of going over the hospital, and was very pleased indeed with all he saw there. The thing that pleased him most was that it was not larger, and he thought the board of management were wise in limiting the size of the hospital to 100 beds. The system of ventilation was perfect, as also was that of drainage. All the appliances were in every way up to date. There was a cheerful air about the whole place which was delightful, and must take away the sting from sickness. Above all there was plenty of air and plenty of sun to warm and purify the air. They had heard of costly failures. No one could call this hospital a costly failure.

Mr. Alan Chambre and Mr. Knox Shaw briefly acknowledged the toast, which was followed by that of "The Ladies" and "The Chairman," proposed in felicitous terms by Dr. Burford.

During the evening the Secretary announced donations amounting to about £7,000, including £52 10s. from the chairman, £1,000 from Captain Cundy (vice-chairman), £100 from Lord Calthorpe, £100 from Sir Henry Tyler (chairman of the House Committee), £200 from Colonel Clifton Brown, £1,000 from a nobleman on condition that the total amount required was subscribed by June in the Hospital Jubilee year; £500, "a jubilee gift from the oldest friend of the hospital;" £500 from "a friend well known to the hospital;" £500 each from Mr. John Carter and Mr. J. H. Houldsworth; and £100 each from Mr. S. B. Brown, Miss Houldsworth, and Mrs. Rylands.

There was an admirable musical programme, carried out under the direction of Mr. Raphael Roche, who was at the piano. The artistes were Miss May Coleman and Señor Guetary (vocalists), Señor Rubio ('cello), and M. Tividar Nachèz (violin).

### CENTRAL LONDON THROAT, NOSE, AND EAR HOSPITAL.

Mr. B. H. Van Tromp, the vice-chairman, presided in the absence of the Chairman, Captain Hutton, at the annual general meeting of the Central London Throat, Nose, and Ear Hospital, on Tuesday, 20th inst. The twenty-fifth report presented by the committee was read by the Secretary, Mr. Richard Kershaw. From this it appeared that 7,948 new cases, involving over 48,000 attendances, were treated during the twelvemonth ended December, 1898, a number largely in excess of any previous year. Unlike most hospitals, the male patients, who numbered 4,059, were in excess of the females, 3,889, the proportion being as 10 to 9, while at the general hospitals the proportion is about 8 to 10. This would appear to show that throat troubles affect the breadwinner in a larger proportion than is the case with other diseases. Many of the patients were children, but very few were old people; the majority ranging between the ages of 20 and 50. The in-patients numbered 315 during the year, the average stay of each being thirteen days. The number of beds in the hospital is sixteen, and of these twelve were occupied daily throughout the year, and the daily average of beds occupied is really higher than it looks, since frequently two of the small wards, containing two beds each, were given up to cases requiring to undergo serious operations, so that only one bed in each was available. The ordinary income amounted to £2,406—just sufficient to leave a margin over the ordinary expenditure, which was £2,244. The only item of extraordinary income was a legacy of £50 from the executors of the late Woolff Joel. The annual subscriptions for the year were £304—rather more than those of last year. The donations were £296, as compared with only £76 in 1897. Steps were taken to bring the Prince of Wales's Hospital Fund stamps prominently before the patients, with the result that enough were sold to make the hospital richer by rather over £22. The committee were, however, greatly disappointed at not receiving a grant from the Fund, more especially as one was made in the first year of distribution. The extraordinary expenditure, on building improvement and interest on bankers' loan, was £138. The balance of revenue over expenditure, £73, was carried to capital account. The total liabilities of the hospital amount to £4,784, and are the cause of much anxiety to the committee; notice has been given that a loan on mortgage on a part of the building must be repaid forthwith. A printed appeal was circulated at Christmas time, but the response was a very limited one; the hope is, however, expressed that friends will help to redeem the debt without the necessity of again borrowing. Attention is called in the report to the large number of medical practitioners signing the attendance book—averaging 1,500 for several years past—as the testimony to the appreciation of the lessons to be learned at the hospital.

In moving the adoption of the report, Mr. Van Tromp expressed regret at the absence of Captain Hutton, who, he said, for twenty-five years had never failed to attend every committee meeting and every annual meeting. He appealed to every subscriber, in view of the wonderful results attained at so small a cost, not only to increase the amount of their own help, but to get their friends and acquaintances to become subscribers likewise.

The re-election of Captain Hutton as chairman and treasurer was carried by acclamation, and a vote of thanks to the medical staff and to the chairman having also been carried, the proceedings terminated.

### OPENING OF NEW HOMES FOR EPILEPTICS AT CHALFONT ST. PETER.

On Friday last week the Duke and Duchess of York visited Chalfont St. Peter to open four new homes at the colony established by the National Society for the Employment of Epileptics. The new homes comprise two for children, both the gift of Mr. J. Passmore Edwards; one for men, the gift of Mr. Frederick Greene; and one for cases requiring special care and treatment, the gift of Mrs. Dearmer. These new homes will in the aggregate increase the existing accommodation of the colony by nearly 100 beds. One of the homes for children has been named after Milton, who resided at the adjacent village of Chalfont St. Giles. The Duke and Duchess of York, accompanied by Sir Charles Cust and Lady Katherine Coke, arrived at the marquee erected for the occasion about three. Amongst those present were Adeline Duchess of Bedford, Sir William and Lady Broadbent, Mr. and Mrs. E. Montefiore Micholls, Mr. and Mrs. Passmore Edwards, Mr. and Mrs. F. Green, Mrs. Dearmer, Mr. Hamilton Hoare, Dr. and Mrs. Buzzard, Dr. and Mrs. Ferrier, Miss Burdon Sanderson, Miss Chadwick, Mr. and Mrs. Pearman, &c. The Royal guests were received by the Chairman (Mr. Montefiore Micholls) and various members of the committee, and the Duchess accepted a bouquet of carnations. After several ladies and gentlemen had been presented, the Chaplain conducted a short religious service.

Mr. Montefiore Micholls, in bidding the visitors welcome, said he saw so many familiar faces that he feared to bore them by the repetition of facts so well known to them. But the occasion was not an ordinary one in the annals of the society. If they had not reached the culminating point in their work, yet they had scaled one of the highest peaks, and time and patience would bring them to the summit. They had now no longer any reason to fear the deep crevasses nor the precipices which discouraged them at the outset. They had homes for men, women, and children, and every man, woman, and child in the kingdom knew that every good work well done to alleviate pain had the sympathy and support of Her Majesty and the Royal Family. They valued the favour and countenance of the Royal Family, for it was only given after wise discrimination. He was happy to announce that the Duke of York had become their president. This was proof that His Royal Highness was satisfied that the work was worthy of His support and sympathy. Mr. Micholls, having sketched the rapid growth and development of the colony, said that before long he hoped that they would possess a convalescent home for epileptics, which would meet one of the greatest needs of the country. At present the fact that people were subject to fits closed the doors of nearly every convalescent institution to them.

The Duke of York, in his interesting address, told the story once more of the beginning in France of the humane treatment of epileptics by healthy, outdoor occupations; how that treatment was next adopted in Germany, and how it had been represented and developed in England since 1894 at Chalfont St. Peter. "It had been estimated," His Royal Highness continued, "that there were 40,000 epileptics in the United Kingdom to whom the common opportunities and objects of life were denied. Requiring in childhood extra care and education, they were neglected and permitted to grow up a burden on their relatives and a source of danger to the community. The colony at Chalfont St. Peter was destined to become an industrial village, wherein these afflicted persons could live and work with the happiest results. Already there was considerable improvement, especially in the younger members of the community. But as yet there was no provision for persons who could afford to pay for their support, and this would, he hoped, be shortly remedied. Referring to statistics, he pointed out how, with increasing numbers, the individual cost of maintenance decreased, and before concluding he referred to the debt of gratitude they owed to the donors of the new homes and to Sir William Broadbent, to whose interest and wise counsel so much of the success of the colony was due.

Sir William Broadbent proposed a vote of thanks to the donors of the new homes, and Mr. Passmore Edwards, in replying, announced that he intended to give a block of buildings for administrative purposes.

The Royal party then adjourned to Milton House, which the Duchess of York inspected and formally pronounced open. Tea was served in the house, and afterwards the Duchess planted a plane tree on the right side and the Duke another on the left of the entrance.

